# Full-Length Transcriptome Sequencing Analysis of Differentially Expressed Genes and Pathways After Treatment of Psoriasis With Oxymatrine

**DOI:** 10.3389/fphar.2022.889493

**Published:** 2022-06-03

**Authors:** Xiaoxiao Xue, Jiayu Yu, Cheng Li, Fang Wang, Yatao Guo, Yongwen Li, Huijuan Shi

**Affiliations:** ^1^ Department of Dermatovenereology, The General Hospital of Ningxia Medical University, Yinchuan, China; ^2^ Dermatological Department, Wuzhong People’s Hospital, Ningxia, China; ^3^ Dermatological Department, Baoji Central Hospital, Shaanxi, China

**Keywords:** psoriasis, oxymatrine, RNA sequencing, differentially expressed genes, enrichment analysis

## Abstract

Psoriasis is a recurrent chronic inflammatory skin disease. Unlike many of the latest psoriasis treatments that only confer limited curative effects and have certain side effects, oxymatrine effectively improves severe plaque psoriasis with mild adverse reactions. Here, we explored the genes and pathways underlying the effects of oxymatrine on psoriasis. Briefly, patients with severe plaque psoriasis were treated with oxymatrine and their lesioned skin samples were sequenced by full-length transcriptomics. Next, the differentially expressed genes (DEGs) in psoriatic lesions were identified and compared in oxymatrine-treated patients and healthy controls, their genes were functionally annotated, and protein–protein interaction network analysis and immunohistochemistry were performed. Both Psoriasis Area and Severity Index (PASI) and Body Surface Area (BSA) scores were recovered significantly from all 16 patients (all *p* < 0.001). The number of DEGs in patients before and after oxymatrine treatment was 4232, and 4105 DEGs were found between the psoriasis group (before oxymatrine treatment) and the normal control group [*p* < 0.01, |log_2_ fold change, (FC)| >1.5]. While most of the DEGs recovered significantly after oxymatrine treatment, only 650 DEGs were observed between the psoriasis group (after oxymatrine treatment) and the normal control group (*p* < 0.01, |log_2_FC|> 1.5). Kyoto Encyclopedia of Genes and Genomes (KEGG) pathway enrichment analysis showed that 64 pathways were significantly activated after oxymatrine treatment (*p* < 0.05). Only 12 pathways were statistically significant between after oxymatrine treatment and the normal control group (*p* < 0 .05). Among all the restored pathways, the improvement of the IL-17 signaling pathway was the most significant (*p* = 1.18E-06). Gene loci of oxymatrine action was assessed by protein interaction analysis on 205 DEGs that were co-expressed in 5 patients before and after oxymatrine treatment (*p* < 0.05, FC > 1.5). After oxymatrine treatment, the expression of two mitosis-related genes namely, cyclin dependent kinase 1 (CDK1) and cyclin B1 (CCNB1), that affect cell proliferation recovered significantly. In light of these results, we conclude that oxymatrine likely alters the abnormal expression of some genes and pathways in psoriasis patients. Multipathway and multitarget therapy can greatly ameliorate abnormalities in genes and pathways and effectively treat psoriasis. Importantly, among the DEGs, the proliferation-related genes, such as CDK1 and CCNB1, are likely important targets for treating psoriasis by oxymatrine. We believe that these findings may lead to a new treatment strategy for psoriasis.

## Introduction

Psoriasis is a recurrent chronic inflammatory skin disease (Seldin, 2015). The currently available oral systemic therapies include retinoids (Chularojanamontri et al., 2019), methotrexate (Raaby et al., 2017), cyclosporine A (Papp et al., 2018), and apremilast (Papp et al., 2015). However, most of these treatments have side effects, and their curative effects are limited. Psoriasis can also be treated with biological agents, which have greatly improved its prognosis (Bai et al., 2019), as they effectively treat moderate to severe psoriasis and have relatively high safety and tolerance levels (Hu et al., 2018). However, biological agents can only be applied under certain conditions, and the potential long-term risks are also unknown (Bai et al., 2019). These agents are associated with an increased risk of infections, though mostly tend to be mild infections. The use of biological agents has certain contraindications and the risks of infection along with long-term usage may potentially lead to tumorigenesis in patients with active pulmonary tuberculosis, advanced congestive heart failure, hepatitis B infection, or demyelination diseases, including multiple sclerosis (Turbeville et al., 2017). Drug resistance and disease recurrence along with unintentional disruption of other pathways are a few shortcomings involved with the usage of biological agents (Reich et al., 2005). Therefore, currently no completely reliable and safe treatment for psoriasis exists, and new treatment methods are urgently needed (Huang et al., 2019).

Psoriasis is often stimulated by infection and certain other factors (Rademaker et al., 2019). It is characterized by excessive proliferation, inflammatory cell infiltration, angiogenesis and other pathological features (Greb et al., 2016) that result in abnormal metabolism and immune regulation imbalance (Gisondi et al., 2018). Oxymatrine is a chemical drug extracted from *Sophora flavescens* (Xia et al., 2011) and has antiproliferative (Shi et al., 2019), anti-inflammatory (Lan et al., 2020), antiviral (Wang et al., 2011), antioxidative (Li et al., 2011) blood lipid–lowering (Shi et al., 2013), and immunoregulatory effects (Xu et al., 2020). Oxymatrine therapy is a single-component drug that exerts multitarget and multi-pathway effects to achieve overall regulation of psoriasis (Lu M. L. et al., 2016). Oxymatrine effectively improves severe plaque psoriasis which is accompanied by mild to adverse reactions (Shi et al., 2019). It is reported that oxymatrine can also inhibit cell proliferation and improve the metabolic syndrome associated with psoriasis (Zhou et al., 2017). However, the mechanisms underlying the efficacy of oxymatrine remain poorly defined and it is imperative to systematically identify them.

In recent years, high-throughput gene expression methods have been widely used to discover pathogenesis and therapeutic response markers (Hrdlickova et al., 2017). Oxford Nanopore Technologies (ONT) sequencing is a unique third-generation sequencing technique that uses electrical signals to identify base sequences (Lu H. et al., 2016). The full-length transcriptome, without interruption, can be used to directly obtain the 5′ to 3′ high-quality full-length sequences of transcripts based on a third-generation sequencing platform (Grabherr et al., 2011). RNA sequencing (RNA-Seq) has been used to measure the expression of genes in patients with psoriasis and healthy individuals in order to identify the major differentially expressed genes (DEGs) in psoriatic lesions (Gudjonsson et al., 2009). The differentially expressed transcripts enriched in the pathogenesis of psoriasis can thus be identified by RNA-Seq. Therefore, full-length transcriptome sequencing can be used to find sensitive and specific biomarkers of psoriasis progression from a large number of genes, screen the genes and pathways most likely involved in the treatment of psoriasis, and clarify the related molecular mechanism. These results may provide a theoretical basis to support clinical application.

In this study, we used full-length transcriptome sequencing to analyze the DEGs in skin tissue from patients with psoriasis before and after oxymatrine treatment and normal controls. Next, we further performed serial analyses, including Gene Ontology (GO) enrichment analysis and Kyoto Encyclopedia of Genes and Genomes (KEGG) pathway analysis. Subsequently, a protein–protein interaction (PPI) network was constructed to identify the gene expression interactions and reveal those genes whose expression may be affected by oxymatrine.

## Materials and Methods

### Subject Enrolment, Treatment, and Sampling

Patients were recruited between July 1 and 15 October 2019, from the General Hospital of Ningxia Medical University. They were diagnosed with psoriasis by clinical and/or histopathological examination in the outpatient department. The inclusion criteria were severe plaque psoriasis, a Psoriasis Area and Severity Index (PASI) score ≥12 (Mattei et al., 2014), a disease course ≥6 months, and at least one previous course of systemic treatment. The exclusion criteria were an allergy to oxymatrine; guttate psoriasis; erythrodermic psoriasis; psoriasis arthropathica; pustular psoriasis; severe liver and kidney damage, mental illness, haematopoietic dysfunction, or other serious organic diseases; treatment with immunosuppressants or high doses of glucocorticoids or retinoids in the past 8 weeks; pregnancy; and lactation. The criteria for removal from the trial were not using the drug, using the drug without following the instructions, stopping before the treatment was complete, or experiencing severe adverse reactions or complications. In this study, patients who met the criteria were recruited sequentially regardless of sex. All participants gave informed, signed consent, and all procedures involving human participants were carried out in accordance with the Declaration of Helsinki. The research project was approved by the Ethics Committee of Ningxia Medical University (clinical trial registration number: ChiCTR-TRC-14004301).

During this period, 18 consecutive patients were diagnosed with severe plaque psoriasis, but 2 patients declined to join the study. Sixteen patients were given 0.6 g/100 ml oxymatrine (Tianqingfuxin; Zhengdatianqing Company Ltd., Jiangsu, China) intravenously once a day for 8 weeks. The dose was based on the recommended dose for the treatment of chronic hepatitis B and has a low level of toxicity (Zhou et al., 2017). Skin biopsies (approximately 0.8 × 1.0 cm) were collected before and after treatment under local anaesthesia. All 16 patients completed oxymatrine treatment and were subjected to biopsies before and after treatment. Psoriatic skin lesions were observed during the course of treatment, and PASI (Mattei et al., 2014) and body surface area (BSA) (Bożek and Reich, 2017) scores were calculated for each patient accordingly. Five patients were randomly selected from the 16 cases using the random number table method. Skin samples were obtained from the healthy skin of patients in the operating room of the Department of Burn and Plastic Surgery. Therefore, we randomly selected 15 samples from 5 patients (pre- and post-treatment for psoriatic skin lesions) and 5 healthy skin samples for full-length transcriptome sequencing.

### Differential Gene Expression Analysis and Statistical Analysis

The DEGs of multiple samples collected from patients with psoriasis and control group were analyzed using the DESeq R package (Gentleman et al., 2004). The *p* values were adjusted using the Benjamini and Hochberg’s approach to control the false discovery rate (FDR). *p* < 0.01 and |log_2_FC| >1.5 as well as *p* < 0.05 and FC > 1.5 were set as the thresholds for significant differential expression in comparisons between paired samples (before versus after oxymatrine treatment, before oxymatrine treatment versus control group and after oxymatrine treatment versus control group). The DEGs were analyzed using the R working environment with the Limma package (Ritchie et al., 2015; Law et al., 2016) from Bioconductor.

### Functional Annotation and Enrichment Analysis

A large number of gene sets were obtained, and common functions or related pathways were investigated through GO node enrichment and KEGG pathway analysis, which are the most common gene analysis methods. Both KEGG and GO terms were defined as statistically significant at *p* < 0.05. To further study the functions of genes, these databases were used. Functional annotation and enrichment analysis were performed using PANTHER (www.pantherdb.org) (Mi et al., 2013; Mi et al., 2019). KEGG pathway analysis was performed to examine the enriched functions in defined biological systems (http://www.genome.ad.jpkegg/pathway.html) (Kanehisa et al., 2012). The distributions of identified DEGs and module genes in the biological process (BP), cellular component (CC) and molecular function (MF) categories were analyzed by GO enrichment utilizing a hypergeometric test (Mi et al., 2019).

### Construction of the PPI Network and Identification of Genes

We searched the online STRING database (http://www.string-db.org/) to identify and predict interactions between genes or proteins. A PPI network of the DEGs was constructed using the haircut algorithm with following threshold conditions: a degree cut-off of 2, a node density cut-off of 0.1, a node score cut-off of 0.2, a K-core value of 2, and a maximum depth of 100. Next, Cytoscape software was used to visualize the PPI network for the DEGs, and its plug-in, Molecular Complex Detection (MCODE) was applied to identify significant modules.

### Immunohistochemistry for CDK1 and CCNB1

IHC of skin tissue was conducted using cyclin dependent kinase 1 (CDK1) (EPR165, Abcam, UK) and cyclin B1 (CCNB1) (Y106, Abcam, UK) antibodies for immunostaining. Skin sections (4 mm) were deparaffinized, rehydrated, blocked and then antigen-repaired. Then, the slides were incubated with rabbit polyclonal antibodies at a dilution of 1:350 at 37°C for 1 h and rinsed with PBS 3 times. Reaction enhancement solution was added, and the slides were incubated at room temperature for 30 min and rinsed with PBS 3 times. Then, the slides were incubated for 30 min with secondary antibodies (Rabbit antibody) at room temperature and rinsed with PBS three times. Finally, 3,3′-Diaminobenzidine was used to develop the color, after which the slides were redyed and sealed. The slides were viewed and photographed under a microscope (Motic, China). The magnification is ×200.The Image-Pro Plus 6.0 immunohistochemical image analysis system was used to analyze the collected images.

### Quantitative Real-Time PCR

The skin lesions of 15 patients were tested using quantitative real-time PCR. A total of 15 RNA samples were tested with GAPDH as the internal reference gene using SYBRGREENI to detect the expression of target genes in the samples. To prepare the cell samples, 1000 μl Trizol was added. Gene expression was quantified according to the 2^–ΔΔCt^ method. Their sequences are listed as follows. CDK1-F: CTT​ATG​CCT​TGG​TCA​GAG​TAA, R: AGA​TGG​CTG​CTA​ATA​AAC​ACT. CCNB1-F: AGC​CAA​GTC​ATG​GAG​AAT​CT, R: GGC​AGC​AAT​CAC​AAG​AAG​AA.

### Western Blotting

Skin lesions of 3 healthy individuals and 3 patients with psoriasis were selected (including before and after treatment). Total protein was extracted from the tissue using RIPA buffer (Epizyme, Shanghai, China) and measured using a BCA kit (Epizyme, Shanghai, China). Protein samples were isolated using 10% SDS-PAGE, transferred to a polyvinylidene fluoride (PVDF) membrane and blocked with 5% skim milk for 2 h. The main antibodies (Anti-CDK1, Rabbit, Cambridge, UK; anti-CCNB1, Rabbit, Abcam, Cambridge, UK) were at dilutions of 1:20000 and 1:50000, respectively. Image density analysis was performed using ImageJ software (NIH Image, Bethesda, MD).

### Statistical Analyses

The data were analyzed using GraphPad Prism 8.0 (GraphPad Software, San Diego, CA, United States)and are presented as the mean ± SD. Student’s t-test was used for comparisons between pre- and post-treatment, and *p* < 0.05 was the threshold for statistical significance.

## Results

### Psoriasis Patient Information and Treatment Effects

**FIGURE 1:**
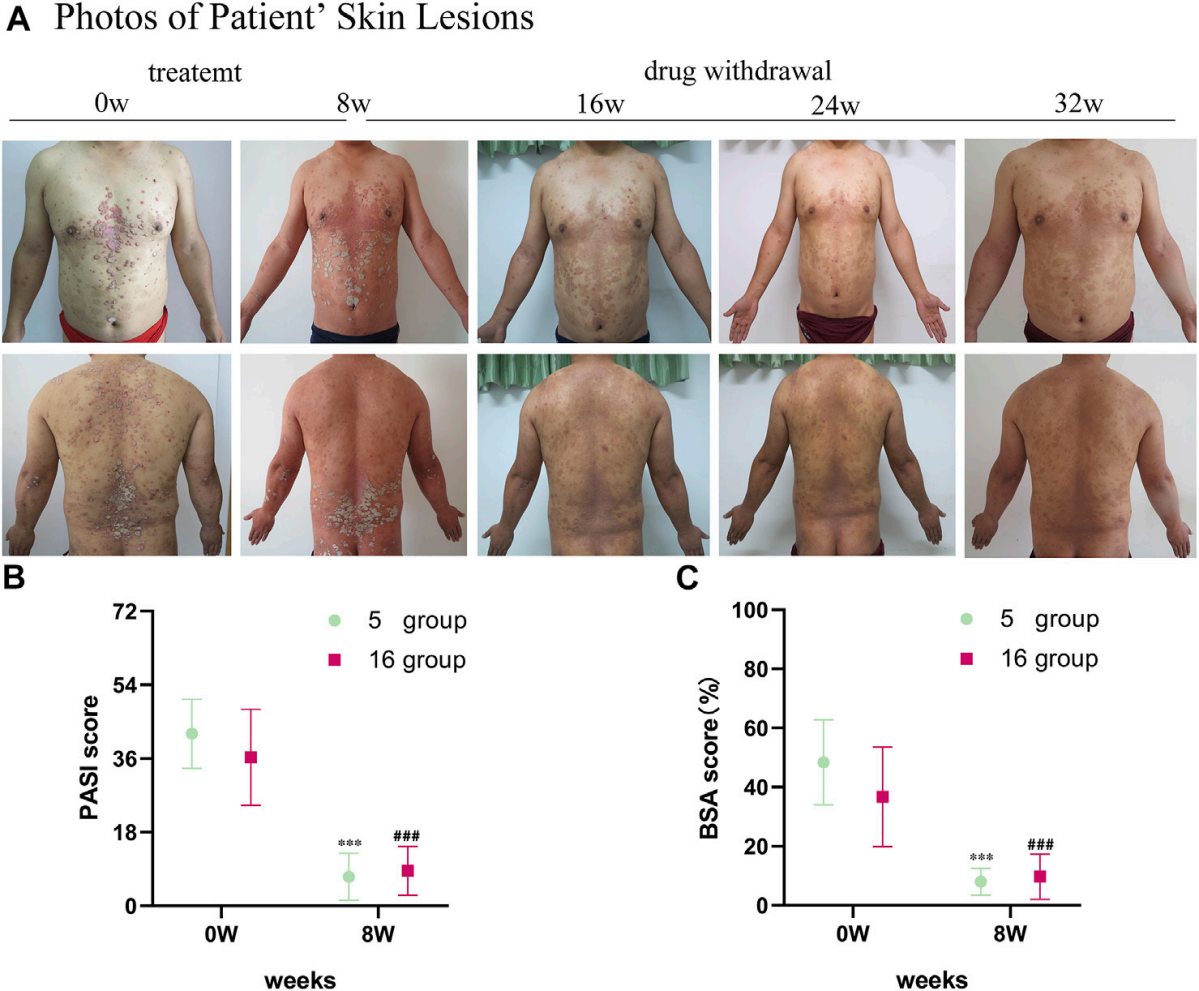
Images of skin lesions and clinical data for patients with oxymatrine treatment. **(A)** Clinical photographs of skin lesions in the oxymatrine group before and 8 weeks after treatment. **(B)** PASI scores and **(C)** BSA scores of patients (before vs. after oxymatrine treatment). *p < 0.05, **p < 0.01,***p < 0.001 vs. before treatment (0 w in “5 group”), ^#^ *p* < 0.05, ^##^ *p* < 0.01 ^###^ *p* < 0.001 vs. before treatment (0 w in “16 group”).

The psoriatic lesions of the patients visibly improved with treatment (Figure 1A). The mean PASI score of 16 patients decreased from the baseline level of 36.29 ± 11.71 to 8.58 ± 5.98 after treatment (*p* < 0.001). The mean BSA score decreased from the baseline level of 36.69 ± 16.82 to 9.69 ± 7.62 after treatment (*p* < 0.001). The average change in PASI score from baseline until the time of evaluation (ΔPASI) was 76%. The mean PASI score of the 5 patients randomly selected for sample collection from the 16 patients with psoriasis was 42.06 ± 8.45, and their mean BSA was 48.4 ± 14.36 at the time of inclusion (baseline). After the 8-week-long treatment with oxymatrine, their PASI scores decreased to 7.10 ± 5.74 (*p* < 0.001), and their BSA scores decreased to 8.00 ± 4.53 (*p* < 0.001). Neither the PASI nor the BSA scores of the 5 patients randomly selected for skin sample collection were significantly different to the larger group of 16 patients (*p* > 0.05). (Figures 1B,C). A total of 15 samples of skin from 5 healthy individuals and 5 patients (before and after treatment of the lesions) were sequenced by full-length transcriptomics (Supplementary Figure S1).

### Expression Profile Analysis With the Full-Length Transcriptome Sequencing Data

**FIGURE 2:**
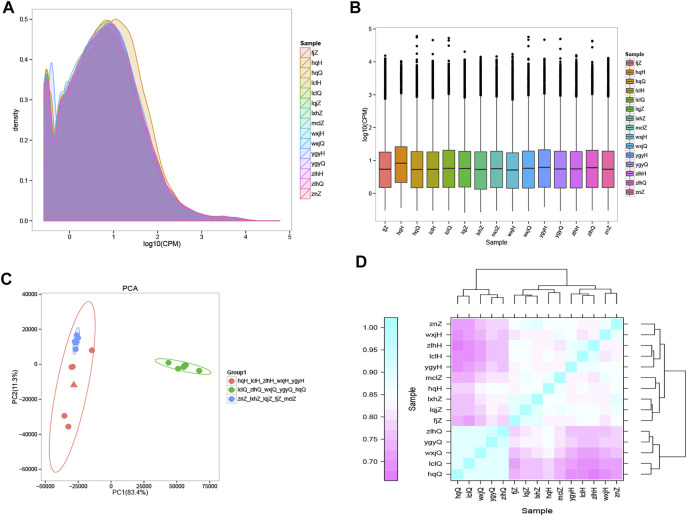
Gene expression distribution The different colours in the contrast map of the gene CPM density distribution of each sample represent different samples, the abscissa represents the logarithm of the CPM of the corresponding sample, and the ordinate represents the probability density. **(A)** The abscissa in the CPM box plot represents the sample, and the ordinate represents the logarithm of the sample expression CPM. The figure shows the expression level of each sample from the point of view of the overall discreteness of the expression quantity. **(B)** PCA map drawn according to the gene expression (CPM) of each sample. Each point represents a sample, each colour represents a sample group, and the ellipse represents the confidence interval of the grouped sample. Orange: psoriasis patients after treatment; green: psoriasis patients before treatment; blue: normal controls. **(C)** Heatmap of the expression correlations in pairs of samples. The abscissa represents the sample, and the ordinate represents the Spearman correlation coefficient r, which was used to evaluate the correlations of biological replicates. **(D)** The expression quantity correlation heat map of pairwise samples: the Abscissa represents different samples; the Spelman correlation coefficient r (Spearman’s Correlation Coefficient) is used as the evaluation index of biological repetition correlation, which is expressed by ordinate.

The results of gene sequencing were qualitatively controlled, and the overall distribution of gene expression in each sample was analyzed (Figure 2A). Transcriptome sequencing was performed using a random sampling process. To ensure that the number of fragments truly reflected the level of gene expression, the number of fragment reads was normalized to the number of mapped reads in the sample. The counts per million (CPM) value was used as an index to measure the level of gene expression (Figure 2B). To further examine the distribution of gene expression levels in a single sample and to clearly compare the overall gene expression levels in different samples, a box diagram was created to show the CPM distribution. Principal component analysis (PCA) (Zhou et al., 2014) was used to analyze the overall distribution trend (Figure 2C) among all 15 samples. It is important to assess the correlations among biological replicates when analyzing transcriptome sequencing data to evaluate not only the repeatability of biological experiments but also the reliability of the DEGs and to assist in the screening of abnormal samples. Spearman’s correlation coefficient was used to evaluate the biological replicate correlations (Figure 2D). Our findings showed that the results obtained with the samples we collected were consistent, reliable, and repeatable. There was a significant difference in distribution between the patients treated with oxymatrine and the same patients before treatment; the distribution of the patients after treatment tended to be consistent with that of the control group. These results indicated that oxymatrine is effective in the treatment of psoriasis, laying a foundation for our follow-up research.

### Identification of DEGs After Oxymatrine Treatment by Full-Length Transcriptome Sequencing

**FIGURE 3:**
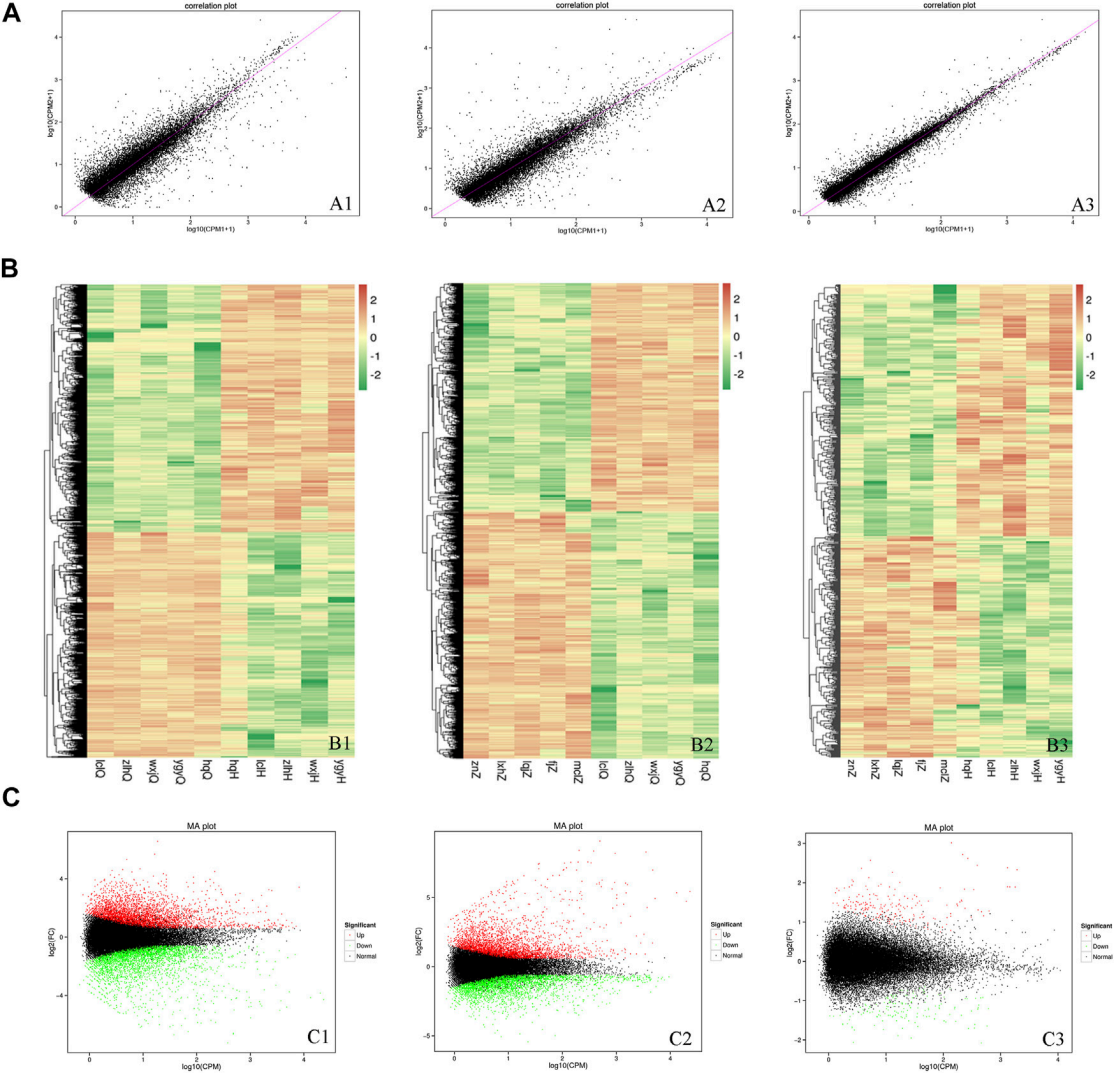
DEGs. **(A)** Map of the expression correlations of all genes in the groups. Each point in the map represents a gene. The abscissa and ordinate correspond to the values of gene expression before and after oxymatrine treatment [log2 (CPM+1)]. **(B)** Heatmap. The abscissa represents the sample name and the sample cluster, and the ordinate represents the DEG and the gene cluster. The different lines represent different genes before vs. after oxymatrine treatment. The colour represents the level of gene expression in the sample [log2(CPM+1E-6)]. **(C)** MA map. Each dot in the MA map of DEGs represents a gene. The abscissa shows the A value [log2(CPM)], which is the logarithm of the mean expression quantity of the two samples. The ordinate shows the M value [log2(FC)], which is the logarithm of the FC in gene expression between the two samples (used to measure the difference in gene expression). Green dots represent downregulated DEGs, red dots represent upregulated DEGs, and black dots represent non-DEGs. A1, B1, C1 (before vs. after oxymatrine treatment). A2, B2, C3 (patients with psoriasis vs. normal controls). A3, B3, C3 (normal controls vs. patients after oxymatrine treatment).

To determine the DEGs, the correlations in gene expression before versus after oxymatrine treatment (r^2^ = 0.5776), before oxymatrine treatment versus in the control group (r^2^ = 0.6241), and after oxymatrine treatment versus in the control group (r^2^ = 0.6724) were analyzed (Figure 3A). In the figure, a greater concentration of points near the diagonal indicates a stronger correlation of gene expression before versus after treatment. The points that deviate from the diagonal represent DEGs. The DEGs are displayed in the heatmaps (before versus after oxymatrine treatment, before oxymatrine treatment versus control group, and after oxymatrine treatment versus control group) (Figure 3B). The MA image shows the DEGs among the three groups. There were 4232 DEGs before versus after oxymatrine treatment [*p* < 0.01, |log_2_ (fold change, FC)| >1.5], of which 2264 genes were upregulated, and 1968 genes were downregulated. Compared with the control group, patients with psoriasis before the treatment exhibited 4105 DEGs, of which 1968 were upregulated and 2137 were downregulated (*p* < 0.01, |log_2_FC| >1.5). Only 650 genes were differentially expressed in oxymatrine-treated patients with psoriasis compared to the control group, of which 346 genes were upregulated and 304 genes were downregulated (*p* < 0.01, |log_2_FC| >1.5) (Figure 3C). The decrease in the number of DEGs from more than 4,000 to 650 indicated that most of the abnormally expressed genes returned to normal after oxymatrine treatment.

### KEGG Pathway Analysis

**FIGURE 4:**
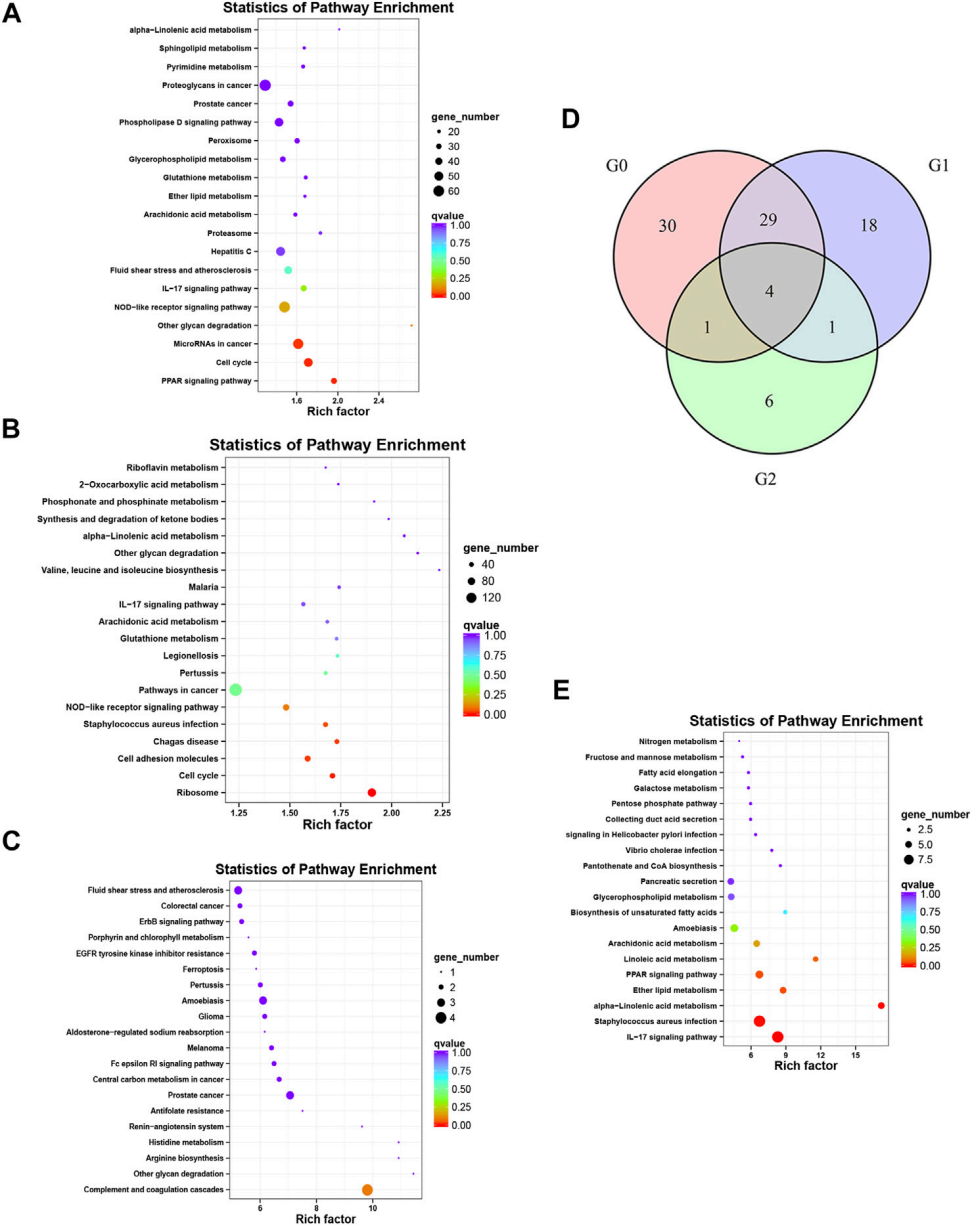
KEGG analysis. Enrichment and distribution map of KEGG pathways of DEGs. Each circle in the map represents a KEGG pathway. The ordinate represents the name of the pathway, and the abscissa (enrichment factor) represents the ratio of the proportion of genes annotated to a pathway among the DEGs to the proportion of genes annotated to a pathway among all genes. **(A)** KEGG pathways for the DEGs before vs. after oxymatrine treatment. **(B)** KEGG pathway for the DEGs in patients with psoriasis vs. normal controls. **(C)** KEGG pathway for the DEGs in oxymatrine-treated patients vs. normal controls. **(D)** Venn map. G0 indicates the number of pathways in the before vs. after oxymatrine treatment comparison. G1 indicates the number of pathways in the after treatment vs. normal control comparison. G2 indicates the number of pathways in the before treatment vs. normal control comparison. **(E)** KEGG pathways of 205 genes.

The enriched KEGG and GO pathways of the DEGs were analyzed and visualized using the R software KEGG pathway enrichment revealed 64 pathways with *p* < 0.05 in the before versus after treatment comparison in psoriatic skin, including 24 pathways related to metabolism; 14 pathways related to cell proliferation and differentiation; 8 pathways related to bacteria, viruses and parasites; 6 pathways related to tumors; 5 pathways related to inflammation and immunity; 4 pathways related to various hormones; and 3 pathways related to angiogenesis (Figure 4A). There were 52 pathways with significant differences in the before versus control comparison (*p* < 0.05), including 13 pathways related to metabolism; 12 pathways related to bacteria, viruses and parasites; 8 pathways related to cell proliferation, differentiation and apoptosis; 8 pathways related to tumors; 8 pathways related to immune response and inflammation; and 3 pathways related to various hormones (Figure 4B). Lastly, KEGG analysis showed that 12 pathways were significantly altered in oxymatrine-treated patients versus the control group (*p* < 0.05), including 4 pathways related to cell proliferation, 3 pathways related to metabolism of various substances, 2 pathways related to infection, 2 pathways related to immune response and inflammation and 1 pathway related to hormones (Figure 4C). Importantly, there were 33 common pathways between the 64 pathways in the before versus after oxymatrine treatment comparison and the 52 pathways in the before versus control group comparison, including 10 pathways related to metabolism of various substances, 7 pathways related to cell proliferation, 6 pathways related to infection, 5 pathways related to tumors, 2 pathways related to hormones, 2 pathways related to immune response and inflammation, and 1 pathway related to angiogenesis. Among the common pathways, 29 (88%) returned to normal after oxymatrine treatment (Figure 4D). Crucially, KEGG analysis revealed no significant alterations in these pathways in the after-treatment versus the control group, indicating that oxymatrine restored these pathways to normal levels. Of the 29 pathways, 28% were related to metabolism, 21% to proliferation, 17% to tumors, 17% to infection, 7% to hormones, 7% to immune response and inflammation, and 3% to angiogenesis. All the DEGs before versus after oxymatrine treatment were used to create a venn diagram (*p* < 0.05 and FC > 1.5), and a total of 205 common DEGs were found (intersection of the diagram). KEGG analysis based on these 205 genes showed that the pathway accounting for the highest number of DEGs was the IL-17 signaling pathway, and most of the genes related to this pathway returned to normal after treatment (Figure 4E). This pathway was also found among the 29 common pathways, indicating that the levels of the associated genes were significantly restored by oxymatrine treatment.

### GO Enrichment Analysis

We conducted GO enrichment analysis of the DEGs in the three groups, and the results included annotated genes belonging to the BP, CC, and MF categories. The results for the before-versus-after oxymatrine treatment comparison with a threshold of *p* < 0.05 revealed 1618 BP pathways, 216 CC pathways, and 382 MF pathways. In the comparison between before oxymatrine treatment and the control group, there were significant differences in 1540, 227, and 356 pathways in the BP, CC, and MF categories, respectively (*p* < 0.05). In the comparison between after oxymatrine treatment and the control group, the results revealed 1469 pathways in the BP category, 221 pathways in the CC category and 354 pathways in the MF category (*p* < 0.05). We also selected the top 5 pathways in the three categories, which are shown in Table 1. Within the top pathways in the BP category (response to drug), the CC category (cytosol) and the MF category (protein binding), there were 63, 486, and 1191 DEGs, respectively, in patients with psoriasis compared with healthy individuals. Before versus after oxymatrine treatment, there were 74, 465, and 1166 DEGs, respectively, in these pathways. Thus, oxymatrine can effectively regulate these genes. Only 11, 48, and 195 genes, respectively, in these three pathways were differentially expressed between the oxymatrine-treated patients (after treatment) and the normal controls, indicating that oxymatrine can change the binding of proteins in the cytosol through the drug response biological process. In the drug response pathway, there were 51 common genes in the patient versus the control group comparison and in the before versus after treatment comparison (51/63, 51/74). There were no significant differences in these 51 genes between the patients after treatment and the control group. Thus, all 51 common genes returned to normal levels after treatment.

**TABLE 1:** GO analysis.

Group	GO Term	*p* Value	Functional Group	Genes
before vs. after oxymatrine treatment	response to drug	7.20E-08	BP	CDK1, CCNB1 (74)
	regulation of cellular response to stress	8.20E-07	BP	(87)
	cellular protein modification process	1.30E-06	BP	CDK1, CCNB1 (383)
	positive regulation of transcription DNA-templated	1.40E-06	BP	(141)
	positive regulation of cell migration	2.90E-06	BP	(70)
	cytosol	1.00E-30	CC	CDK1, CCNB1 (486)
	extracellular exosome	9.70E-27	CC	(435)
	cytoplasm	1.40E-25	CC	CDK1, CCNB1 (1277)
	nucleoplasm	4.50E-20	CC	CDK1, CCNB1 (333)
	perinuclear region of cytoplasm	2.10E-09	CC	(91)
	protein binding	1.00E-30	MF	CDK1, CCNB1 (1191)
	ATP binding	1.70E-11	MF	(176)
	RNA binding	7.50E-07	MF	(148)
	protein serine/threonine kinase activity	1.40E-05	MF	CDK1, CCNB1 (53)
	protein kinase binding	2.30E-05	MF	(71)
before oxymatrine treatment vs. normal control group	ageing	3.60E-07	BP	(56)
	nuclear-transcribed mRNA catabolic process, nonsense-mediated decay	5.20E-07	BP	(35)
	response to drug	5.60E-07	BP	CDK1, CCNB1 (63)
	SRP-dependent cotranslational protein targeting to membrane	7.90E-07	BP	(29)
	establishment of organelle localization	1.10E-06	BP	(49)
	cytosol	1.00E-30	CC	CDK1, CCNB1 (465)
	cytoplasm	3.40E-27	CC	CDK1, CCNB1 (1231)
	extracellular exosome	3.50E-27	CC	(412)
	nucleoplasm	1.50E-22	CC	CDK1, CCNB1 (333)
	perinuclear region of cytoplasm	3.30E-11	CC	(94)
	protein binding	1.00E-30	MF	CDK1, CCNB1 (1166)
	ATP binding	7.50E-10	MF	(163)
	RNA binding	5.80E-07	MF	(147)
	protein homodimerization activity	9.90E-06	MF	(94)
	ubiquitin protein ligase binding	1.80E-05	MF	(38)
after oxymatrine treatment vs. normal control group	aorta development	6.60E-06	BP	(1)
	response to drug	1.90E-05	BP	(11)
	response to oestrogen	2.60E-05	BP	(3)
	cellular protein modification process	3.30E-05	BP	(42)
	pancreas development	3.80E-05	BP	(1)
	cytosol	5.50E-25	CC	(48)
	cytoplasm	9.30E-20	CC	(174)
	nucleoplasm	7.50E-18	CC	(26)
	extracellular exosome	7.70E-17	CC	(87)
	perinuclear region of cytoplasm	1.70E-08	CC	(15)
	protein binding	1.00E-30	MF	(195)
	ATP binding	2.30E-12	MF	(21)
	alcohol binding	1.60E-06	MF	(5)
	growth factor binding	1.30E-05	MF	(10)
	RNA binding	1.60E-05	MF	(7)

GO, enrichment included terms in the BP, CC, and MF, categories. We selected the top 5 pathways in the three categories. It shows that the gene changes in the pathway before and after treatment, especially CDK1 and CCNB1.

GO, gene ontology; BP, biological process; CC, cellular component; MF, molecular function. “Genes” indicates number of genes and whether CDK1 and CCNB1 are included.

From the above results, we found that the number of differentially activated pathways was the highest in the before versus after oxymatrine treatment comparison, indicating that many genes and gene pathways changed after oxymatrine treatment. Oxymatrine treatment stimulated a drug response; however, the cell stress response, protein modification, transcription, and other processes were also involved in the process by which oxymatrine ameliorated psoriasis. Most of these reactions occurred in organelles such as the cytosol, extracellular exosomes, and cytoplasm. However, between the treated patients (after treatment) and the normal controls, there were 149 fewer differentially activated pathways. This decrease in the number of GO pathways indicated that some genes and their pathways returned to normal. This finding also suggested that oxymatrine treatment improved the levels of multiple genes and pathways.

Notably, in the GO analysis of the top 5 terms for the before versus after oxymatrine treatment comparison, the genes Cyclin B-dependent kinase 1 (CDK1) and CCNB1 were both associated with the following terms: response to drug, cellular protein modification process, cytosol, cytoplasm, nucleoplasm, protein binding and protein serine/threonine kinase activity.

### Construction of a PPI Network Based on DEGs

**FIGURE 5:**
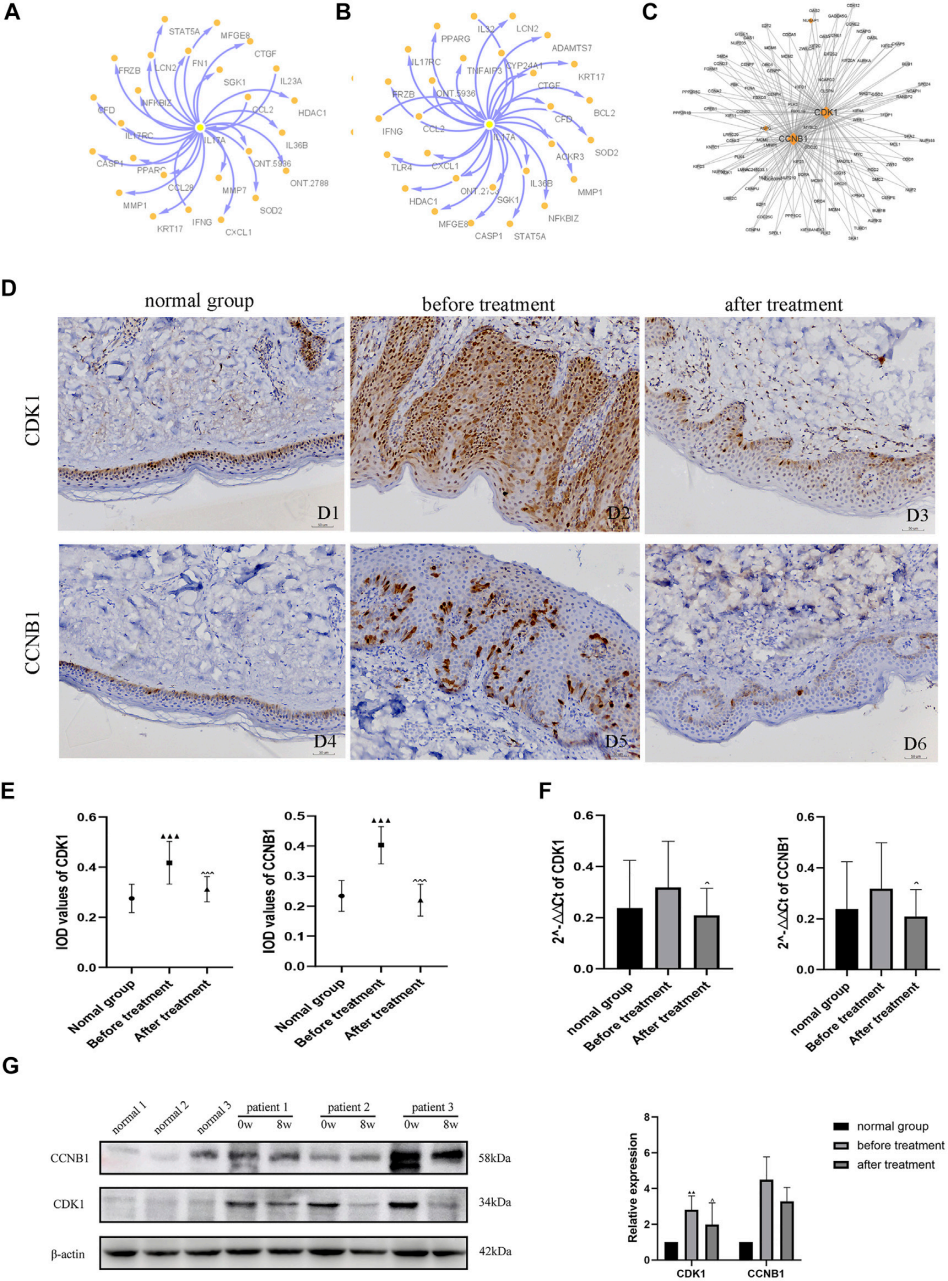
PPI network analysis and IHC. **(A)** Coexpression network of IL-17A (before vs. after oxymatrine treatment). **(B)** Coexpression network of IL-17A (before oxymatrine treatment vs. normal control group). **(C)** PPI network of 205 common DEGs. **(D)** (D1-D3) IHC staining for CDK1, (D4-D6) IHC staining for CCNB1. D1, D4: normal control group; D2, D5: before oxymatrine treatment; D3, D6: after oxymatrine treatment. **(E)**Integrated optical density (IOD) values of CDK1 and CCNB1. **(F)** qRT-PCR of CDK1, CCNB1 (^▲^ *p* < 0.05,^▲▲^ *p* < 0.01,^▲▲▲^ *p* < 0.001 vs. normal control group; ^*p* < 0.05, ^^*p* < 0.01,^^^*p* < 0.001 vs. before oxymatrine treatment). **(G)** Western blot of CDK1 and CCNB1 protein.

To explore the interactions among proteins in patients with psoriasis-related diseases, we constructed a PPI network. First, we performed PPI analysis on the core gene IL-17A in the IL-17 pathway, which had the most significant differential enrichment (*p* = 1.18E-06) (Figures 5A,B). Protein interaction analysis was carried out on the 205 DEGs co-expressed in 5 patients before versus after oxymatrine treatment to identify the gene loci of oxymatrine action (*p* < 0.05, FC > 1.5). From the resulting PPI network, the protein with the highest degree was selected. Among the 205 genes, there were 26 genes that interacted with each other. The interaction between the CDK1 and CCNB1 proteins ranked first. CDK1 and CCNB1 are related to mitosis and affect cell proliferation. After oxymatrine treatment, the expression of these two genes recovered significantly (Figure 5C).

### Effects of Oxymatrine on CDK1 and CCNB1 in the Skin of Patients With Psoriasis as Detected by Immunohistochemistry, Quantitative Real-Time PCR and Western Blotting

In patients with psoriasis, the expression of CDK1 was widely distributed in all layers of the epidermis. The cytoplasm and nuclei of the cells in each layer were also stained. The expression of CDK1 in the lesions of patients before treatment was significantly stronger than that in healthy skin (*p* < 0.001). Importantly, after oxymatrine treatment, the expression of CDK1 in the skin tissue of patients decreased significantly (*p* < 0.001). There was no significant difference between the after-treatment and the control groups (*p* > 0.05), indicating that oxymatrine treatment restored the expression of CDK1 in skin lesions to normal levels (Figure 5D).

Low expression levels of CCNB1 were found in the nucleus and cytoplasm in healthy skin, mainly in the basal cell layer of the epidermis and part of the hair follicle infundibulum. The expression of CCNB1 in psoriatic lesions and all layers of the epidermis was significantly higher than that in healthy skin (*p* < 0.001). However, the expression of CCNB1 in the skin tissues of patients treated with oxymatrine was significantly decreased (*p* < 0.001), such that there was no significant difference between patients treated with oxymatrine (after treatment) and the control group (*p* > 0.05). The results showed that oxymatrine treatment decreased the expression of CDK1 and CCNB1, restoring it to the same level as that in normal tissue (Figure 5E).

The expression of CDK1 and CCNB1 were analyzed by qRT-PCR. Following treatment with oxymatrine, the expression of CDK1 and CCNB1 decreased significantly compared to the levels before treatment (*p* < 0.05). These results suggested that oxymatrine does reduce the expression of CDK1 and CCNB1 in patients’ skin (Figure 5F).

Subsequently, using western blot analysis. We detected the expression of these two genes in skin tissue of patients before and after oxymatrine treatment. CDK1 expression was increased in patients with psoriasis (versus control group) (*p* < 0.01), but decreased significantly after oxymatrine treatment (vs. before treatment) (*p* < 0.05). This trend also occurred in the expression of the CCNB1 gene (Figure 5G).

## Discussion

Psoriasis is a chronic inflammatory skin disease (Griffiths and Barker, 2007) that is difficult to treat because these skin lesions are persistent and recurrent (Kaushik and Lebwohl, 2019). Although targeted therapy for psoriasis with biological agents has high efficacy, it interferes with immune homeostasis. Additionally, with increasing treatment time, related problems such as infection, autoimmune reactions, malignant tumors, drug failure and disease recurrence may appear (Armstrong and Read, 2020). The factors that induce psoriasis are extremely complex, and many of the current psoriasis treatments have limitations. Thus, it is imperative to improve our understanding of the mechanisms underlying treatments and to explore new and more effective treatments.

Our results showed that the PASI improvement rate was 76% after 8 weeks of oxymatrine treatment, indicating that it significantly improved the skin lesions of psoriasis patients. This result is in agreement with that of a previous study by Zhou et al*.*, that oxymatrine is effective in the treatment of severe plaque psoriasis Zhou et al. (2017). In our study, we used full-length transcriptome sequencing to screen 4105 DEGs between psoriatic lesions and normal skin (|log_2_FC| >1.5, *p* < 0.01). This number of DEGs was similar to that reported by Li et al., who identified 3577 DEGs by RNA-Seq between psoriatic lesions and normal skin (Li et al., 2014). Following oxymatrine treatment, physical symptoms as indicated by PASI and BSA scores significantly improved, and the expression of more than 4232 genes in psoriasis patients was significantly altered. Additionally, only 650 genes were abnormally expressed, meaning their levels remained different between patients following oxymatrine treatment and the normal controls. Further, after oxymatrine treatment, although the expression of most of the genes returned to normal levels, the expression of some genes did not fully recover. These results showed that oxymatrine regulates the majority of the abnormally expressed genes in patients with psoriasis.

Psoriasis is often characterized by excessive proliferation, abnormal differentiation, angiogenesis and inflammatory cell infiltration triggered by infection and trauma (Greb et al., 2016). Patients often suffer from disrupted metabolism, abnormal hormone levels and immune system disorders (Sommer et al., 2006). In our study, the KEGG analysis results for the comparison between psoriasis patients before treatment with oxymatrine and normal controls showed that 52 pathways related to metabolism, proliferation, infection and immunity were significantly altered. This finding indicates that compared with normal subjects, patients with psoriasis have abnormal expression of molecules in these pathways, which is consistent with the literature on the pathogenesis of psoriasis (Greb et al., 2016). KEGG analysis also revealed that oxymatrine treatment ameliorated the alterations in a total of 64 pathways. Thirty-three pathways were shared between the patients with psoriasis and the control group. Of these 33 pathways, 29 (88%) pathways exhibited significantly improved alterations and returned to the same levels of activation as in the normal control group. The KEGG analysis from another study on patients with psoriasis treated with the adalimumab drug showed that only the cytokine–cytokine receptor interaction pathway was significantly enriched (Langkilde et al., 2016). In contrast, our enrichment analysis showed that alterations in many pathways related to metabolism, immune inflammation, cell proliferation and infection were greatly improved following oxymatrine treatment. In addition, KEGG analysis indicated that the IL-17 signaling pathway was the most significantly enriched after oxymatrine treatment. IL-17 increased the expression of antimicrobial peptides, including β-defensin regulates the expression of 8 genes in the IL-17 signaling pathway including S100A family proteins (S100A7, S100A9, S000A7A, CXCL1, CXCL8, DEFB4A, DEFB4B, and LCN2) (Lynde et al., 2014). Therefore, unlike the single-target therapy achieved by biological agents, oxymatrine improves psoriasis by altering not only the important IL-17 pathway but also affects the inflammatory pathway, the infection pathway, and the metabolic pathway, achieving multipathway and multitarget therapy. This finding provides a putative explanation for how some studies have shown that oxymatrine can treat and inhibit the recurrence of plaque psoriasis (Zhou et al., 2017). Unlike many previous studies, that have focused on the role of oxymatrine specific pathways and genes (Li et al., 2018; Gan et al., 2020; Xiang et al., 2020), our study provides a macroscopic perspective of oxymatrine action in psoriasis.

GO analysis found that in the response to the drug, 51 genes were shared between the patients versus the normal control group and the before versus after treatment comparison. After treatment, all 51 genes returned to normal levels. Among the 26 gene interactions, the interaction between CDK1 and CCNB1 (hub genes) was the most significant. In the normal control group versus after treatment comparison, the two DEGs CDK1 and CCNB1 were not enriched in any GO pathways. CDK1 promotes mitosis (Melero et al., 2018) and CCNB1 regulates the binding of maturation-promoting factor (MPF) and CDK1 to drive the phase transition of the cell cycle at the G2/M phase junction and initiate mitosis (Frouin et al., 2005). A similar role of cyclins in psoriasis has been reported by Miracco et al. (2000). Related research has found that the cell cycle is significantly shortened in patients with psoriasis and that high expression of CDK1 and CCNB1 promotes keratinocyte proliferation observed in psoriasis (Ni and Lai, 2020). This is consistent with the results of our GO analysis, IHC, qRT-PCR. And western blot analysis. Furthermore, oxymatrine is reported to inhibit epidermal proliferation and apoptosis (Shi et al., 2019). In view of these results, we speculate that oxymatrine can induce cell cycle arrest and apoptosis through the genes CDK1 and CCNB1 to inhibit cell activity and proliferation. CDK1 and CCNB1 may be important targets of oxymatrine in the treatment of psoriasis.

This study had some limitations. For example, the sample size of the designed dataset was relatively small, and the study was a single-centre study. Despite these limitations, we conclude that oxymatrine can greatly improve the expression of many abnormally expressed genes and pathways.

In summary, our results show that oxymatrine significantly reduced the erythema scales of the patients’ skin lesions and improves the abnormal state of expression of genes and inflammation and proliferation pathways. Whether this occurs via IL-17 signal pathway, or CDK1, CCNB1 and other proliferation-related genes is not clear. Oxymatrine treatment regulated several pathways related to metabolism, tumors, and infection and by regulating cell proliferation, apoptosis, inflammatory factor levels, and hormone levels. Based on these results, we speculate that the regulatory effect of oxymatrine on psoriasis is not unilateral but rather that multiple signaling pathways participate in the comprehensive effects of oxymatrine on pathological factors of psoriasis directly or indirectly. Moreover, the changes in DEGs represented by CDK1 and CCNB1 expression may be important biomarkers for oxymatrine efficacy in the treatment of psoriasis. These findings may facilitate the development of a new and effective strategy for psoriasis treatment.

## Data Availability

The datasets presented in this study can be found in online repositories. The names of the repository/repositories and accession number(s) can be found below: ncbi.nlm.nih.gov; PRJNA815711.

## Supplementary Material

Supplementary Figure S1 The flow chart of the study of this article.
